# Development of Consumption

**Published:** 1893-09

**Authors:** 


### Editorial, Etc.

DEVELOPMENT OF CONSUMPTION.—Doctor Pepper, in a clinical lecture recently delivered at the University of Pennsylvania, took occasion to note the influence of surroundings with the development of consumption in certain individuals.

Some persons have an undoubted tendency to this disease, which tendency they have inherited from one or the other parent. If both parents have suffered from the disease, the tendency is more pronounced.

It must not be supposed that every one inheriting a consumptive tendency succumbs to it. It is during the years preceding maturity that the danger of poor, unhygienic surroundings is greatest, but "if such persons can be kept strong until their forms have developed, they may become the very strongest of the strong."

The conditions are favorable for the development of consumption only when "the system gets 'run down;' then follows a cold, a catarrh, the bacilli of tuberculosis become lodged in the mucous membrane, invade the tissues, and spread."

Speaking of the influence of dusty occupations and of the dust which is inhaled from the streets and in travelling conveyances, the speaker said: "There is no one here who has not had tubercle-bacilli enter his air-passages, but there must be some resisting power which has made it impossible for the organisms to gain entrance into the system, and which has prevented them from spreading."

When the surroundings of an individual are unhygienic, the occupation uncongenial and depressing. or such as to prevent sufficient sleep, the health often becomes impaired. Then the strongest constitution may offer but little resistance to the ingress of consumption. It is safe to say that every case of consumption is first ingrafted under just such circumstances.

"Let a person task his strength, let a growing child become weedy, lank and below weight, and the system relaxed, and there is developed a field where tuberculosis, if implanted, will spread."

Persons in whose families this disease has existed should recognize the fact that there may be a constitutional lack of resistance in themselves. Fatigue and excesses of all kinds should be avoided. As much of life as is possible should be spent in the open air, and the rules of health, which are applicable to all alike, should be carefully observed.—*The Youth's Companion.*

---

### Monthly Summary.

